# Anatomical distribution of cerebral microbleeds and intracerebral hemorrhage in vertebrobasilar dolichoectasia

**DOI:** 10.1371/journal.pone.0196149

**Published:** 2018-04-19

**Authors:** Alex Förster, Ralf Wenz, Máté Elöd Maros, Johannes Böhme, Mansour Al-Zghloul, Angelika Alonso, Christoph Groden, Holger Wenz

**Affiliations:** 1 Department of Neuroradiology, University Hospital Mannheim, University of Heidelberg, Mannheim, Germany; 2 Department of Life Sciences, Faculty of Natural Sciences, Imperial College London, London, United Kingdom; 3 Department of Neurology, University Hospital Mannheim, University of Heidelberg, Mannheim, Germany; Universitatsklinikum Freiburg, GERMANY

## Abstract

**Objectives:**

Vertebrobasilar dolichoectasia (VBD) is a dilatative arteriopathy associated with intracerebral hemorrhage. In the present study, we sought to evaluate the frequency and anatomical distribution of cerebral microbleeds (cMBs) and intracerebral hemorrhage (ICH) in VBD.

**Methods:**

From a MRI database 94 VBD patients were identified and analyzed with special emphasis on cMBs and ICH on T2*-weighted gradient echo images (GRE) in relation to the established diagnostic MRI criteria of VBD (diameter, height, and lateral position). cMBs/ICH location was categorized into anterior/posterior circulation. Clinical information like demographic details, clinical symptoms, and comorbidities were abstracted from the case records. An extensive modelling approach using generalized linear mixed-effects models was used.

**Results:**

Overall, 79 (84.0%) patients (mean age 72.1±10.0 years, 74.7% male) with a standard stroke MRI protocol including T2*-weighted images were included in the analysis. cMBs were observed in 38/79 (48.1%) patients, ranging from 1 to 84 cMBs per patient. In the posterior circulation cMBs were observed more frequently (34/38 (89.5%)) in comparison to the anterior circulation (24/38 (63.2%)). cMBs were observed in the thalamus in 20/38 (52.6%), hippocampus in 1/38 (2.6%), occipital lobe in 18/38 (47.4%), pons in 6/38 (15.8%), medulla oblongata in 2/38 (5.2%), and cerebellum in 14/38 (36.8%) patients. ICH was observed in only 6/79 (7.6%) patients. There were significantly more cMBs in the posterior- (NCMBs-PC = 1.717, 95%CI: 1.336–2.208, p = 0.0315) than in the anterior circulation.

Logistic regression model showed a significant positive effect of clinical symptoms such as ischemic, TIA and hemorrhagic stroke on the presence of cMBs (OR = 3.34, 95%CI [2.0–5.57], p = 0.0184; ndf = 78, AIC = 107.51).

General linear model showed that clinical symptoms have a highly significant effect on the number of cMBs (N = 2.78, 95%CI [2.51–3.07], p<2*10–16; ndf = 78, AIC = 1218).

**Conclusion:**

cMBs and ICH may be observed in the anterior and posterior circulation in VBD but they occur more frequently in the posterior circulation. Most common anatomical locations of cMBs in VBD were the thalamus, occipital lobe and cerebellum. This posterior dominance of cMBs and ICH in VBD might reflect a specific underlying vascular pathology.

## Introduction

Vertebrobasilar dolichoectasia (VBD, syn. megadolichobasilar artery or anomaly, and fusiform aneurysm) is a relatively rare arteriopathy with elongation, widening, and tortuosity of the vertebral arteries (VA) and the basilar artery (BA)[1]. An association with connective tissue disorders like Ehlers-Danlos syndrome or Marfan syndrome, [2,3] polycystic kidney disease, [4,5] Fabry’s disease, [6,7] AIDS, [8] neurofibromatosis type 1, [9,10] atherosclerosis and chronic dissection [11] has been reported. Risk factors associated with VBD comprise age, male sex, arterial hypertension, smoking habit, and coronary artery disease [12]. While VBD may be asymptomatic, it can also be the cause of transient ischemic attack (TIA) or stroke in the posterior circulation. Furthermore, vertebrobasilar dolichoectasia may be the cause of subarachnoid or intracerebral hemorrhage (SAH, ICH), or lead to compression of the cranial nerves or brainstem [13–17]. Recently, an association of cerebral microbleeds (cMBs) and VBD has been reported with a predominant localization of cMBs in the vertebrobasilar territory [18]. Cerebral microbleeds are defined as small, homogeneous, round foci of low signal intensity on gradient echo T2*-weighted magnetic resonance imaging without corresponding hypo- or hyperintesity on conventional MRI [19]. Etiologies underlying cMBs include several arteriopathies such as hypertensive arteriopathy, cerebral amyloidangiopathy, Moyamoya disease, and cerebral autosomal dominant arteriopathy with subcortical infarcts and leukoencephalopathy (CADASIL) [20]. Furthermore, presence of cMBs is associated with an increased risk of ICH [21,22].

### Aims

In the present, study we sought to evaluate (1) the frequency of cMBs and ICH in VBD with special emphasis on the (2) number and distribution of cMBs in the posterior circulation territory, and (3) a possible correlation of the presence and number of cMBs with the diameter, height, and lateral displacement of the BA.

## Methods

This retrospective single-center study was approved by the institutional review board (IRB) “Ethikkommission II der Universität Heidelberg, Medizinische Fakultät Mannheim”. IRB did not require written informed consent, hence none was obtained. Nonetheless, in keeping with the IRB guidelines, exceeding care was taken for the relevant data to be anonymized and de-identified prior to the analysis.

### Patients

From a MRI report database (2004–2016), we identified 94 patients with VBD. Patients were identified using the following synonyms of vertebrobasilar dolichoectasia, such as megadolichobasilar artery or anomaly as well as fusiform aneurysm, basilar artery dilation and basilar ectasia. Of these, 79 patients underwent a standard stroke MRI protocol including time-of-flight-(TOF)-MR angiography (MRA) and gradient echo T2*-weighted images. These patients formed the study population and were studied with regard to demographic details, clinical symptoms, and comorbidities as abstracted from the case records. This study has been approved by the local institutional review board.

### Magnetic resonance imaging

Magnetic resonance imaging was performed on a 1.5-T MR system (Magnetom Sonata and Magnetom Avanto, Siemens Medical Solutions, Erlangen) or a 3-T MR system (Magnetom Trio, Siemens Medical Solutions, Erlangen). A standardized stroke MRI protocol was used in all patients including (1) transverse, coronal and sagittal localizing sequences followed by transverse oblique contiguous images with a slice thickness of 5 mm aligned with the inferior borders of the corpus callosum (applied on sequences 2 to 6); (2) T1-weighted images; (3) T2-weighted images; (4) diffusion-weighted images (DWI); (5) fluid attenuated inversion recovery (FLAIR) images; (6) gradient echo T2*-weighted images; and (7) a TOF-MRA with a slice thickness of 1 mm. Parameters of gradient echo T2*-weighted images are displayed in Table 1.

**Table 1:** Sequence parameters of T2*-weighted images at the department’s MRI scanners.

Parameters	MRI scanner		
	1.5-T Siemens	1.5-T Siemens	3-T Siemens
	Sonata	Avanto	Trio
**FOV**	240	230	230
**Number of slices**	24	24	24
**Flip angle**	19	20	20
**ST**	5	5	5
**TR**	670	814	620
**TE**	16	26	20

FOV = field of view (mm x mm), ST = slice thickness (mm), TR = repetition time (ms), TE = echo time (ms).

### MRI analysis

**Fig 1:**
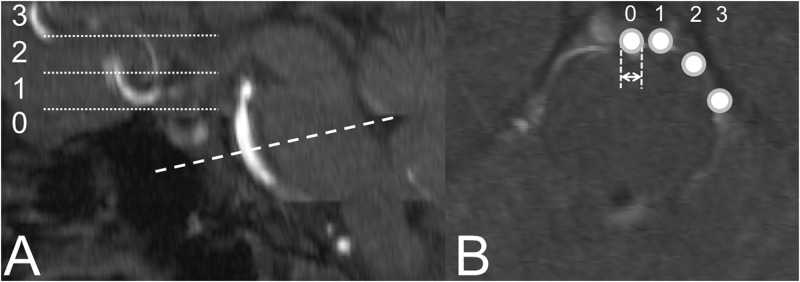
Schematic illustration of the magnetic resonance imaging diagnostic criteria for vertebrobasilar dolichoectasia. A. Height of basilar artery bifurcation. B. Diameter at the mid-pons level (double arrow) and lateral position of the basilar artery.

OsiriX v.5.0.2 a multidimensional image navigation and display software was used for TOF-MRA to reconstruct 2D multiplanar reconstruction images in axial, coronal, and sagittal planes[23]. The degree of vertebrobasilar dolichoectasia according to the established MRI criteria [24] was determined on reconstructed images in axial plane parallel to the orbitomeatal line as described previously [25]: in brief, the diameter of the BA at the mid-pons level (≤ 4.5 mm, > 4.5 mm), height of BA bifurcation (at/below dorsum sellae, within suprasellar cistern, at level of third ventricle floor, indenting and elevating third ventricle floor), and lateral displacement (midline, midline or questionably off midline, definitely displaced to the side, reaching the cerebellopontine angle) were assessed. For further analyses the lateral positions of the BA on the right and the left side in its course (R→L) according to the MRI criteria were graded 0 (no lateral displacement) 1 (0→1/1→0), 2 (1→1), 3 (0→2/2→0), 4 (1→2/2→1), 5 (2→2), 6 (0→3/3→0), 7 (1→3/3→1), 8 (2→3/3→2), and 9 (3→3) as established earlier [25,26]. Likewise, the height of the top of the BA was graded 0 (at/below dorsum sellae), 1 (within suprasellar cistern), 2 (at the level of third ventricle floor), 3 (indenting and elevating third ventricle floor) as established earlier [25,26]. To illustrate how the lateral displacement and the height of the BA was classified see Fig 1.

In terms of detection of anterior circulation arteriomegaly, we refer to the MRI criteria of Brinjikji et al.[27].

**Fig 2:**
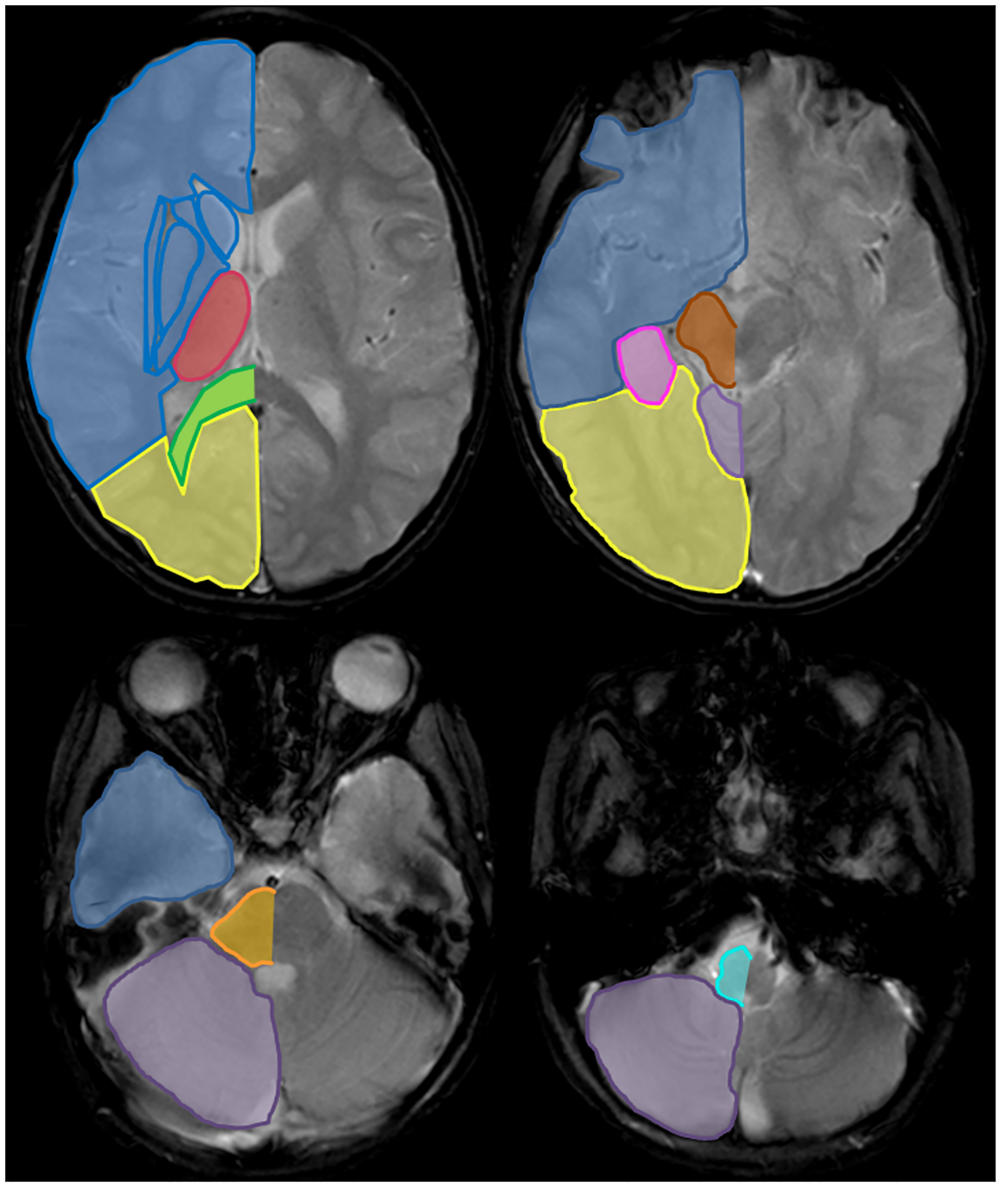
The distribution of cerebral microbleeds in vertebrobasilar dolichoectasia were noted according to the to the maps by Tatu et al. [27,28] Supratentorial: Anterior circulation (blue), and posterior circulation including occipital lobe (yellow), thalamus (red), splenium (green), and hippocampus (pink). Infratentorial: Brainstem, including mesencephalon (brown), pons (orange), and medulla oblongata (turquoise), and cerebellum (violet).

Cerebral microbleeds were diagnosed on gradient echo T2*-weighted images according to the proposed guidelines[19]. In order to distinguish cMBs from potential differential diagnosis findings on gradient echo T2*-weighted images were compared to the other MR images. Localization of cMBs in the anterior and posterior circulation was determined according to the maps by Tatu et al.[28,29] and the topographical distribution of cMBs in the posterior circulation categorized in (1) posterior cerebral artery (PCA) territory, (2) mesencephalon, (3) pons, (4) medulla oblongata, and (5) cerebellum. For a schematic illustration see Fig 2. In the PCA territory, cMBs were further categorized in (1) thalamus, (2) splenium, (3) hippocampus, (4) occipital lobe, and (5) mesencephalon.

Magnetic resonance images were analyzed by two independent raters (A.F. and H.W., both with eight years of experience in neuroimaging) blinded to the clinical information. Cases with discrepancies were re-reviewed by both readers and discussed until a consensus was reached.

### Statistical analysis

Statistical analyses were performed either using Statistical Product and Service Solutions (SPSS) statistics for Windows (Release 17.0; SPSS, Chicago, IL, USA) or the R statistical programming environment using the lme4 package [30] explicitly designed for fitting linear mixed-effects models.

Descriptive data was analyzed by use of Chi-square tests and the Mann-Whitney U Test as appropriate. Comparison between patients with or without cMBs were performed using Chi-square tests and the Mann-Whitney U Test as appropriate with a 0.05 level of significance.

To evaluate the effect of clinical symptoms (ischemic/TIA and hemorrhagic stroke) on the presence and number of cMBs we performed a descriptive analysis with a logistic regression model; for the investigation of clinical symptoms on the number of cMBs we performed a general linear model due to the classical Poisson distribution of number of cMBs.

In terms of the frequency of cMBs in the anterior and posterior territory we performed a Jonckheere-Terpstra trend followed by an extensive modelling approach using generalized linear mixed-effects models accounting for the Poisson distributed response variable.

The final model setup with the best model fit (i.e. lowest AIC) was a nested random effects model which used an intercept varying among patients and among vascular territories within patients. Thus, we could account for individual patient characteristics and for correlations if both vascular territories (anterior and posterior circulation) were affected within the same patient. This model (AIC = 492.6) included fixed effect terms for age, gender, the diameter of the BA, the height of BA head and vascular territories.

The inclusion of laterality of basilar artery (ordinal factor with 9 levels) was not possible as such models did not converge. For such analyses probably, a larger cohort is needed to be able to estimate the covariance matrix.

## Results

### Baseline characteristics and clinical presentation

In the final analysis 79 patients with VBD were included (mean age 72.1±10.0 years, 74.7% male). In the majority of patients (74.7%) VBD was an incidental finding. Overall, there were 18/79 (22.8%) patients with transient ischemic attack (TIA) or acute ischemic stroke, 6/79 (7.9%) patients with intracerebral hemorrhage, and 2/79 (2.5%) patients with subarachnoid hemorrhage. Of these, 15/79 (19.0%) cases with transient ischemic attack (TIA) or acute ischemic stroke, and 2/79 (2.5%) cases with subarachnoid hemorrhage were possibly related to VBD. Furthermore, 2/79 (2.5%) patients had clinical symptoms due to brainstem compression, and 1 (1.3%) patient due to hydrocephalus caused by VBD. Details on demographics and cerebrovascular risk factors are given in Table 2.

**Table 2:** Details on demographics and cerebrovascular risk factors in vertebrobasilar dolichoectasia patients with and without cMBs.

n (%), unless noted	All patients, n = 79	Patients with cMBs, n = 38	Patients without cMBs, n = 41	p value
**Age, years, mean (± SD)**	72.1 (±10.0)	72.8 (±9.5)	71.4 (±10.4)	0.55
**Male**	59 (74.7)	29 (76.3)	30 (73.2)	0.75
**Cerebrovascular risk factors**				
** Arterial hypertension**	68 (86.1)	33 (86.8)	35 (85.4)	0.85
** Diabetes mellitus**	11 (13.9)	6 (15.8)	5 (12.2)	0.65
** Hyperlipidemia**	26 (32.9)	13 (34.2)	13 (31.7)	0.81
** Previous TIA/stroke**	9 (11.4)	4 (10.5)	5 (12.2)	0.82
** Coronary heart disease**	11 (13.9)	3 (7.9)	8 (19.5)	0.14
** Renal insufficiency**	8 (10.1)	4 (10.5)	4 (9.8)	0.91
** Cigarette smoking**	4 (5.1)	2 (5.3)	2 (4.9)	0.94
**Associated pathology**				
** TIA/Stroke**	18 (22.8)	10 (26.3)	8 (19.5)	0.47
** Intracerebral hemorrhage**	6 (7.6)	6 (15.8)	0	**0.008**
** Subarachnoid hemorrhage**	2 (2.5)	1 (2.6)	1 (2.4)	0.96
**Criteria for VBD**				
** BA diameter, mean (SD)**	6.6 (4.7)	7.4 (5.5)	6.0 (3.8)	0.19
** BA lateral position, median (IQR)**	5 (4–8)	5 (4–8)	5 (4–8)	0.72
** BA height, median (IQR)**	2 (1–2)	2 (1–3)	2 (1–2)	0.25

TIA = transient ischemic attack, VBD = vertebrobasilar dolichoectasia, BA = basilar artery, cMBs = cerebral microbleeds

Clinical symptoms with a possible relationship to cMBs could be registered in all (yes/no 27/67) patients and cases with available cMBs (yes/no 25/54), only.

### MRI and TOF-MRA analysis

#### Vertebrobasilar dolichoectasia

On TOF-MRA the mean diameter at the mid-pons level was 6.6 ± 4.7 mm. The height of the BA bifurcation was graded 1 in 32 (40.5%), 2 in 28 (35.4%), and 3 in 19 (24.1%) patients. The noted most lateral positions of the BA on the right and the left side in its course (R→L) was graded 0→1/1→0 in 1/79 (1.3%), 1→1 in 6/79 (7.6%), 0→2/2→0 in 7/79 (8.9%), 1→2/2→1 in 15/79 (19.0%), 2→2 in 12/79 (15.2%), 0→3/3→0 in 5/79 (6.3%), 1→3/3→1 in 6/79 (7.6%), 2→3/3→2 in 20/79 (25.3%), and 3→3 in 7/79 (8.9%) patients.

### Incidence and distribution of cMBs

#### Baseline characteristics

In total, we detected 395 cMBs (mean number 10.4±16.4, range 1–84) within 38/79 (48.1%) patients with VBD. For a comparison of patients with and without cMBs see Table 2. Of the cMBs, 181 were located in the anterior circulation, and 214 in the posterior circulation. In the posterior circulation cMBs were observed in more frequently (34/38 (89.5%)) in comparison to the anterior circulation (24/38 (63.2%)). In 14/38 (36.8%) patients cMBs were exclusively found in the posterior circulation. The remaining patients (51.9%) had no cMBs.

**Fig 3:**
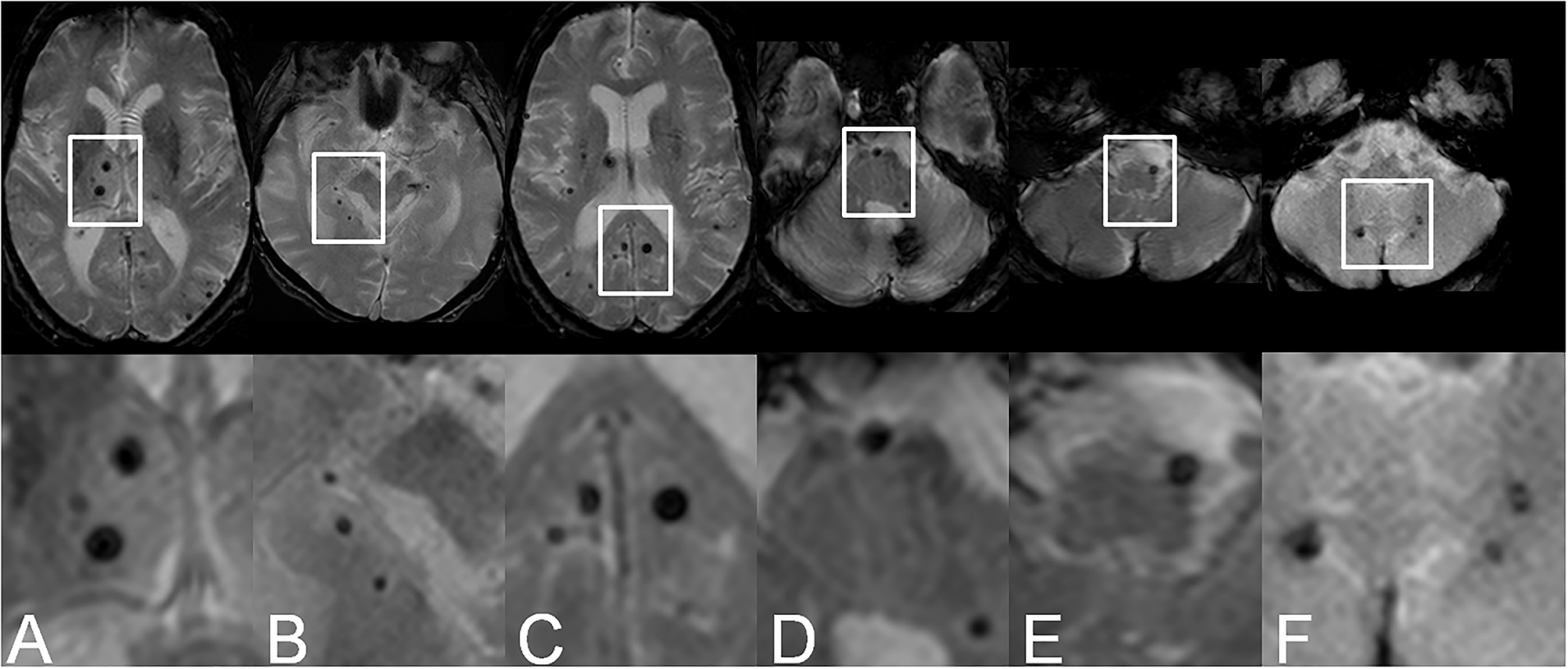
Examples of cMBs in the posterior circulation on T2*-weigthed images. A. Thalamus. B. Hippocampus. C. Occipital lobe. D. Pons. E. Medulla oblongata. F. Cerebellum.

In the posterior circulation cMBs were observed in the thalamus in 20/38 (52.6%), in the hippocampus in 1/38 (2.6%), in the occipital lobe in 18/38 (47.4%), in the pons in 6/38 (15.8%), in the medulla oblongata in 2/38 (5.2%), and in the cerebellum in 14/38 (36.8%) patients. For examples see Fig 3. In the mesencephalon and the splenium no cMBs could be detected. Distribution of cMBs in the posterior circulation is shown in Table 3.

**Table 3:** Distribution of cMBs in the posterior circulation in patients with vertebrobasilar dolichoectasia.

Localization	VBD patients, n (%)	cMBs, range
**Occipital lobe**	18 (47.4%)	1–28
**Hippocampus**	1 (2.6%)	4
**Thalamus**	20 (52.6%)	1–15
**Splenium**	0	0
**Mesencephalon**	0	0
**Pons**	6 (15.8%)	1–2
**Medulla oblongata**	2 (5.2%)	1–2
**Cerebellum**	14 (36.8%)	1–6

VBD = vertebrobasilar dolichoectasia, cMBSs = cerebral microbleeds

**Fig 4:**
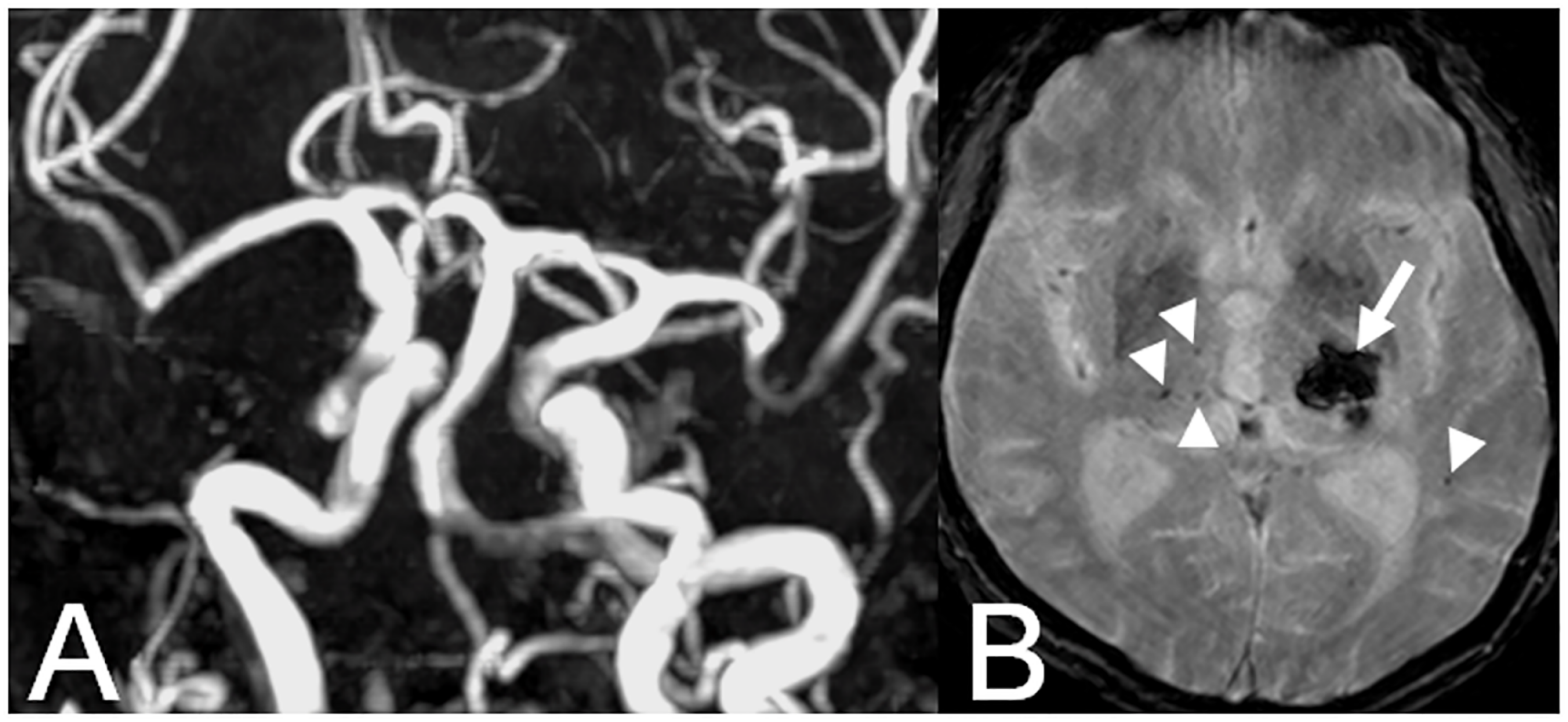
Example of a patient with A. VBD on TOF-MRA and B. associated intracerebral hemorrhage in the thalamus (arrow) as well as cMBs (arrow heads) in the posterior circulation on T2*-weigthed images.

Intracerebral hemorrhage was observed in 6/79 (7.6%) patients. Of these, 5/79 (6.3%) were located in the posterior circulation: in the thalamus in 4/5 (80%), and in the occipital lobe in 1/5 (20%) patients. For an example see Fig 4.

#### Territorial distribution of cMBs

The Jonckheere-Terpstra trend test (with number of 1000 permutations), showed a significant trend (JT = 357, p = 0.002) towards if both vascular territories were affected.

In terms of the nested random effects model, the intercept was almost significantly different from 0 (NcMBs = 0.00931, 95%CI: 0.000799–0.108, p = 0.0568) supporting our random intercept approach. Age (p = 0.427), diameter of the BA (p = 0.394), and BA height (p = 0.931) showed no relevant association with the number of cMBs. Males had significant more cMBs than females (NCMBs-Male = 2.256, 95%CI: 1.555–3.273, p = 0.0288). Comparing vascular territories showed that there were significantly more cMBs in the posterior- (NCMBs-PC = 1.717, 95%CI: 1.336–2.208, p = 0.0315) than in the anterior circulation. The model showed no signs of overdispersion using an approximative Chi-square test (Chisq = 26.00, ratio: 0.1733, resid.df: 150, p = 1.0).

There were only weak or negligible positive correlations of fixed effects of posterior circulation territory with gender (0.247), diameter of BA (0.014) and negligible negative correlation with age (-0.001), height of BA (-0.015).

#### Arteriomegaly and cMBs of the anterior circulation

In total, 8 (8.5%) out of the 94 patients presented with an arteriomegaly in the anterior and posterior circulation. In case of the patients with T2* weighted scans, 7 patients (8.9%) had an arteriomegaly in the anterior and posterior circulation. Of these 7 patients, one patient (1.3%) only had cMBs in the anterior circulation, while one other patient (1.3%) had cMBs in the anterior and posterior circulation. The other 5 patients (6.3%) did not have cMBs in either location.

#### Correlation of the presence and number of cMBs with the extent of VBD

In general, in the anterior as well as in the posterior circulation, neither presence nor number of cMBs showed a significant stronger correlation with the BA diameter, the height of BA bifurcation, or the lateral position of the BA.

#### Effect of clinical symptoms on the presence of cMBs

The logistic regression model showed a significant positive effect of clinical symptoms on the presence of cMBs (OR = 3.34, 95%CI [2.0–5.57], p = 0.0184; ndf = 78, AIC = 107.51); (AIC: = Akaike information criterion).

#### Effect of clinical symptoms on the number of cMBs

General linear model showed that clinical symptoms has a highly significant effect on the number of cMBs (N = 2.78, 95%CI [2.51–3.07], p<2*10–16; ndf = 78, AIC = 1218).

## Discussion

In the present study, we investigated the incidence and detailed anatomical distribution of cMB in a large cohort of patients with VBD. Primarily, cMBs are radiological finding describing homogeneous hypointense lesions in normal or near normal brain tissue on susceptibility-weighted sequences after extravasation of blood [19]. Also, cMBs are commonly thought to be a manifestation of small vessel disease [31] and there exists a strong association between cMBs and basilar artery dilation [32]. Detection rate of cMBs depends on the sequence used, field strength and echo time (TE) [19,33,34]. Recently, a higher prevalence of cMBs located predominantly in the posterior circulation has been reported and a more specific underlying vascular pathology was hypothesized in patients with VBD [18].

The present study describes three essential findings regarding cMBs in patients with VBD: (1) cMBs can be found primarily in the posterior circulation, and (2) most frequently in the thalamus, occipital lobe, and cerebellum, and (3) ICH associated with VBD can be found in corresponding localizations.

Prevalence of cMBs in patients with VBD in the present study was approximately 48% which is much higher in comparison to the healthy population. The Rotterdam Scan Study, (based on 1,062 persons) for example, showed an age-specific prevalence of cMBs of 5.4% for the age group of 60–69 years, rising to 23.3% for the age group of 80–97 years [35]. In particular, in terms of cMB localization, we showed that cMBs were more frequently observed in the posterior circulation. While the first can be at least partly attributed to the shared risk factors in patients with VBD and cMBs, the latter might indicate a specific underlying pathology of small vessels in the posterior circulation in patients with VBD.

Another study that looked into the location of microbleeds in conjuction with arterial dolichectasia was done by Thijs et al. [31]. They could show that microbleeds were more common in the brain stem, deep regions, and in corticosubcortical areas in patients with arterial dolichectasia compared to those without. By distinguishing between the posterior and anterior circulation, we further build on findings of Thijs et al. [31] by showing that cMBs are primarily in the posterior circulation, and most frequently in the thalamus, occipital lobe, and cerebellum. However, in our study the statistical significance was only modest when comparing anterior and posterior circulation. Their study together with our study gives a more complete picture of the distribution of microbleeds in vertebrobasilar dolichoectasia.

Macroscopic and microscopic anatomy of the posterior circulation differs significantly from the anterior circulation with regard to the supplying vessels (the vertebral arteries (VA) merge and form the single midline basilar artery), the higher frequency of congenital vessel variations (e.g. hypoplasia of the VA, fetal origin of the PCA), and the large variety of supplied anatomical structures (neocortex, allocortex, thalamus, brainstem, and cerebellum)[36]. Furthermore, differences in the prevalence of atherosclerosis risk factors [37] as well as differences in pathological changes such as vessel thickening, elastin loss, and concentric intimal thickening [38] between anterior and posterior circulation have been demonstrated only recently. Comparable differences in pathological changes between the anterior and posterior circulation have not been reported for the smaller vessels yet. However; since small vessel disease is more common in the posterior circulation[37,39] such differences might be assumed as likely. Finally, general differences in stroke etiologies [37,39] and complications in the clinical course such as secondary intracerebral hemorrhage have been reported[40].

In general, cMBs are associated with significantly higher risks of intracerebral bleeding, e.g. in cerebral amyloid angiopathy or hypertensive arteriopathy [21,22]. In VBD, subarachnoid hemorrhage and intracerebral hemorrhage have been reported in approximately one fifth of VBD patients [16]. In this study, intracerebral hemorrhage was located in vascular territories supplied by vessels emanating from dolichoectatic parent vessels, whereas all subarachnoid hemorrhages were limited to one or more cisterns around the brain stem [16]. Most interestingly, 5 (6.3%) patients within our cohort had intracerebral hemorrhage in the posterior circulation, and all of these patients had cMBs. This finding is of importance as it is well established that microbleeds are associated with increased likelihoods of intracranial hemorrhages [41]. Therefore, our findings of increased amounts of microbleeds in the posterior circulation in patients with VBD might hence indicate that this patient population has a higher likelihood of developing intracerebral bleedings in the posterior circulation.

The presents study has some limitations. First, this is a retrospective study of moderate size. However, to our knowledge, this is the first larger study focusing exclusively on cMBs in VBD patients. Second, the study was performed with different MRI scanners and different imaging sequences. However, the MRI sequences, especially the T2* weighted sequences, have been customized for optimal comparability in daily clinical routine and consequently are generally comparable. Thirdly, because vertebrobasilar dolichoectasia has several synonyms and milder forms of VBD might be classified differently some patients have been missed in the database search. We tried to circumvent this by searching as many synonyms as possible.

Fourthly, when keeping in mind that the classical treatment for VBD is dual antiplatelet therapy, it would be very interesting to investigate the relationship between antiplatelet therapy and cMBs. However, because of the nature of this retrospective study, the design did not allow us to make confident conclusion about this relationship. Future studies should prospectively be designed to make such investigations possible. Finally, the hospital-based retrospective study design might cause several types of bias and statistical errors such as selection bias, sample bias, or image-based selection bias.

In patients with vertebrobasilar dolichoectasia cMBs and ICH may be observed in the anterior and posterior circulation but they occur more frequently in the posterior circulation. Most common anatomical locations of cMBs in VBD were the thalamus, occipital lobe and cerebellum. This posterior dominance of cMBs and ICH in VBD might reflect a specific underlying vascular pathology.

## Supporting information

S1 Table Table includes data for fitting linear mixed-effects models such as age, diameter of basilar artery (BA), clinical symptoms (such as ischemic, TIA and hemorrhagic stroke), height of BA, laterality of BA, number of cerebral microbleeds (cMB) and territory of cMBs. (XLSX)
